# Comparative Efficacies of Collagen-Based 3D Printed PCL/PLGA/β-TCP Composite Block Bone Grafts and Biphasic Calcium Phosphate Bone Substitute for Bone Regeneration

**DOI:** 10.3390/ma10040421

**Published:** 2017-04-17

**Authors:** Kyoung-Sub Hwang, Jae-Won Choi, Jae-Hun Kim, Ho Yun Chung, Songwan Jin, Jin-Hyung Shim, Won-Soo Yun, Chang-Mo Jeong, Jung-Bo Huh

**Affiliations:** 1 Department of Prosthodontics, Dental Research Institute, Institute of Translational Dental Sciences, BK21 PLUS Project, School of Dentistry, Pusan National University, Yangsan 50612, Korea; ksubi42@naver.com (K.-S.H.); won9180@hanmail.net (J.-W.C.); cmjeong@pusan.ac.kr (C.-M.J.); 2 Department of Mechanical System Engineering, Graduate School of Knowledge-Based Technology and Energy, Korea Polytechnic University, 237 Sangidaehak-Ro, Siheung-Si, Gyeonggi-Do 15073, Korea; kjh0019@kpu.ac.kr; 3 Department of Plastic and Reconstructive Surgery, Kyungpook National University, School of Medicine, 130 Dongdeok-ro, Jung-gu, Daegu 41944, Korea; hy-chung@knu.ac.kr; 4 Department of Mechanical Engineering, Korea Polytechnic University, 237 Sangidaehak-Ro, Siheung-Si, Gyeonggi-Do 15073, Korea; songwan@kpu.ac.kr (S.J.); happyshim@kpu.ac.kr (J.-H.S.); wsyun@kpu.ac.kr (W.-S.Y.)

**Keywords:** PCL/PLGA/β-TCP, 3D printing, collagen, bone grafts, BCP

## Abstract

The purpose of this study was to compare bone regeneration and space maintaining ability of three-dimensional (3D) printed bone grafts with conventional biphasic calcium phosphate (BCP). After mixing polycaprolactone (PCL), poly (lactic-co-glycolic acid) (PLGA), and β-tricalcium phosphate (β-TCP) in a 4:4:2 ratio, PCL/PLGA/β-TCP particulate bone grafts were fabricated using 3D printing technology. Fabricated particulate bone grafts were mixed with atelocollagen to produce collagen-based PCL/PLGA/β-TCP composite block bone grafts. After formation of calvarial defects 8 mm in diameter, PCL/PLGA/β-TCP composite block bone grafts and BCP were implanted into bone defects of 32 rats. Although PCL/PLGA/β-TCP composite block bone grafts were not superior in bone regeneration ability compared to BCP, the results showed relatively similar performance. Furthermore, PCL/PLGA/β-TCP composite block bone grafts showed better ability to maintain bone defects and to support barrier membranes than BCP. Therefore, within the limitations of this study, PCL/PLGA/β-TCP composite block bone grafts could be considered as an alternative to synthetic bone grafts available for clinical use.

## 1. Introduction

A sufficient amount of residual bone in an edentulous alveolar ridge is required for successful implant treatment [1]. The use of bone grafts has been popularized in bone defect reconstruction due to the development of tissue engineering and regenerative medicine [2]. Bone grafts are classified into autogenous bone grafts, allografts, xenografts, and synthetic bone grafts depending on donor tissues and materials [3]. The autogenous bone grafts which are the most ideal bone grafts for restoring bone defects have all the properties necessary for new bone growth—including osteogenecity, osteoconductivity, and osteoinductivity—but some studies have reported their weaknesses such as additional surgery, donor site morbidity, and limited availability [4,5,6]. To overcome the limitations of autogenous bone grafts, allograft and xenograft materials have been developed. However, allografts and xenografts remain at risk for infection due to donor tissue [4,5,6]. Therefore, a lot of interest has been focused on methods and materials for the production of synthetic bone grafts that can be produced on large scales and utilized without causing immune reactions [4,5,6,7].

It is crucial to determine the optimal size and shape of bone grafts in consideration of space maintenance for bone regeneration, support of periosteum or barrier membrane, and anatomical structure of bone defects [8]. In the meantime, synthetic bone grafts have been produced in particle form or block form depending on their size and shape [9,10]. Particle form bone grafts manifest excellent manageability and rapid re-vascularization. However, it is difficult to maintain their shape as the particles often disperse or even disappear [9,11]. On the other hand, block form bone grafts can be effectively used on relatively large bone defects due to their excellent mechanical strength and shape-retaining ability, but they display some disadvantages: the healing time is prolonged due to a delayed re-vascularization, and they require complicated techniques while having structural problems [9,11,12]. Recently, it has become possible to produce a desired specified shape by using a solid freeform fabrication (SFF) technique, which is a three-dimensional (3D) printing technique where bone grafts are manufactured in the appropriate size, shape, and pore geometry, ranging from the micrometer unit to bone defect size [13,14]. Several studies have confirmed that bone grafts made by SFF technique are easily maintain shape and possess excellent interconnectivity between pores, thereby increasing cell penetration and increasing nutrient circulation and oxygen supply [5,15,16].

The available materials for SFF technique are polymers such as polycaprolactone (PCL), poly (lactic-co-glycolic acid) (PLGA), polylactic acid, and polyglycolic acid [8,15,16]. In a recent study, PCL/PLGA/β-TCP block bone grafts, which were fabricated using SFF technique in a bone defect scale, were introduced by adding β-tricalcium phosphate (β-TCP) to a mixture of PCL and PLGA [5]. This study showed that the material had excellent mechanical strength and biocompatibility and that it was excellent in space maintenance ability and useful for new bone regeneration [5]. However, CT images are essentially required for customized bone graft scaffolding. Moreover, it is necessary to spend significant amounts of time and effort when the defect size is large or complicated.

Therefore, in order to overcome these problems, this study has developed a micro-sized PCL/PLGA/β-TCP particulate bone grafts through SFF technique, mixed with a collagen matrix in order to fabricate a customized-composite block form. These composite block form graft materials present excellent plasticity, enabling to cutting or compaction into a desired shape. Furthermore, the collagen matrix located among the bone grafts prevents them from disappearing while improving structural stability [12]. The purpose of this study was to develop block bone graft that was composed with collagen and PCL/PLGA/β-TCP composite particles which were 3D-printed with SFF technique, and to evaluate space maintenance ability and new bone formation capability, comparing to biphasic calcium phosphate (BCP) which is widely used as calcium phosphate ceramics in dental practice.

## 2. Materials and Methods

### 2.1. Preparation of Blended PCL/PLGA/β-TCP

PCL (19561-500G, MW 43,000–50,000; Polysciences Inc., Warrington, PA, USA), PLGA (430471-5G, MW 50,000–75,000; Sigma-Aldrich, St. Louis, MO, USA), and β-TCP (average diameter 100 nm; Berkeley Advanced Biomaterials Inc., Berkeley, CA, USA) were admixed using a thermal melting process [15,16,17]. Briefly, granular PCL (0.4 g) and PLGA (0.4 g) were melted and blended in a glass container at 160 °C for 10 min, and then β-TCP (0.2 g) powder was added to the molten PCL and PLGA and then was mixed for 5 min [15].

### 2.2. Fabrication of PCL/PLGA/β-TCP Particulate Bone Grafts Using 3D Printing Technology

**Figure 1:**
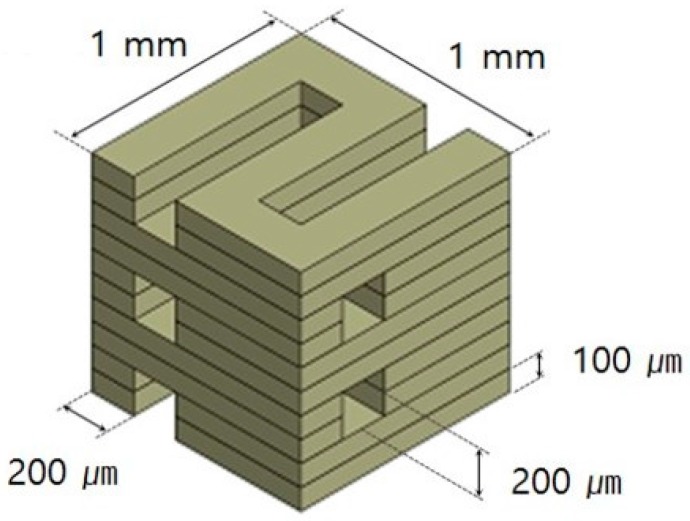
Schematic drawing of PCL/PLGA/β-TCP particulate bone grafts. The line width, pore size, and line height were fixed at 200, 200, and 100 μm, respectively.

The PCL/PLGA/β-TCP mix was transferred into a 10 mL steel syringe attached to an extrusion-based 3D printing system and dispensed at 135 °C [15]. Cubical PCL/PLGA/β-TCP particulate bone grafts (1 × 1 × 1 mm^3^) were fabricated. The line width, pore size, and line height were fixed at 200, 200, and 100 μm, respectively. Therefore, the calculated porosity was approximately 32%, and the pores were fully interconnected (Figure 1).

### 2.3. Fabrication of Collagen-Based PCL/PLGA/β-TCP Block Bone Grafts

**Figure 2:**
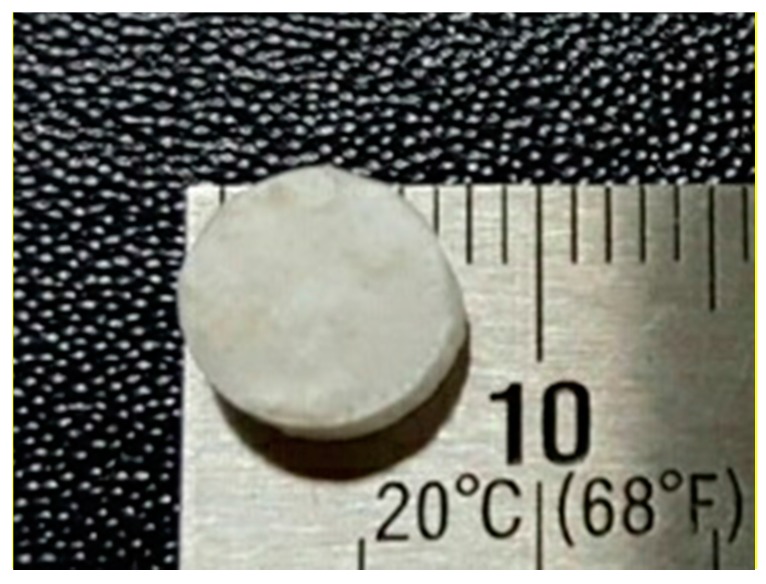
Photographs of PCL/PLGA/β-TCP composite block bone grafts, 8 mm in diameter and 2 mm in height.

Fabricated particulate bone grafts were mixed with 3% atelocollagen (TheraFill^®^, Sewon Cellontech, Seoul, Korea), and the mixed solution was poured into a PDMS mold to make a definite shape. The molded collagen was incubated at 37 °C for 15 min followed by deep freezing (6 h) and freeze drying (12 h), and cross-linked by immersing in ethanol/water (90% *v*/*v*) co-solvent containing 50 mM of 1-ethyl-3-(3-dimethyaminopropyl) carbodiimide (EDC) and 20 mM of N-hydroxysuccinimide (NHS) for 24 h at room temperature. The cross-linked collagen block was freeze-dried again with the same conditions described above. The block form specimens with a diameter of 8 mm and a height of 2 mm were fabricated (Figure 2).

### 2.4. Components Analysis for PCL/PLGA/β-TCP Particulate Bone Grafts

The components ratio of PCL and PLGA in PCL/PLGA/β-TCP particulate bone grafts were measured with FT-IR (Fourier transform infrared spectroscopy). 1 g of fabricated PCL/PLGA/β-TCP particulate bone grafts were dissolved in 100 g of 99.5% chloroform (C0584, Samchun Pure Chemical Co., Pyeongtaek-si, Korea). The solution was filtered with circulation aspirator system (DH.WEV0003S, Daihan scientific, Wonjoo-si, Korea) and 0.45 μm filter papers (MTF045047H, CHMLAB Group, Barcelona, Spain). After filtration, the spectrum of solution was recorded using a FT-IR (Frontier MIR, PerkinElmer Inc., Waltham, MA, USA) by KBr pellet technique (L1271192, PerkinElmer Inc.).

The β-TCP in PCL/PLGA/β-TCP particulate bone grafts were confirmed with energy dispersive X-ray spectroscopy (EDS). The filtered powders were well collected in petri dishes, rinsed with pure water three times and dried until fully dried. After the drying process, the powder was sputter coated with platinum for measuring the calcium and phosphorus ratio using an EDS equipment (NORAN system 7, Noran Instruments Inc., Middleton, WI, USA).

### 2.5. Scanning Electron Microscope (SEM) Observation

The dried PCL/PLGA/β-TCP particulate bone grafts were sputter–coated with platinum and observed by a field emission scanning electron microscope (FE-SEM) (S-4700, Hitachi, Tokyo, Japan) operated at an accelerating voltage of 10 kV [14].

### 2.6. Experimental Animals and Surgical Procedure

Thirty-two male Sprague-Dawley rats (250-300 g in weight) were used, and all rats were isolated and kept in standard laboratory conditions. All experimental procedures were carried out using an animal selection management method and a surgical protocol approved by the Pusan National University Animal Experimental Ethics Committee (PNU-2015-0919).

**Figure 3:**
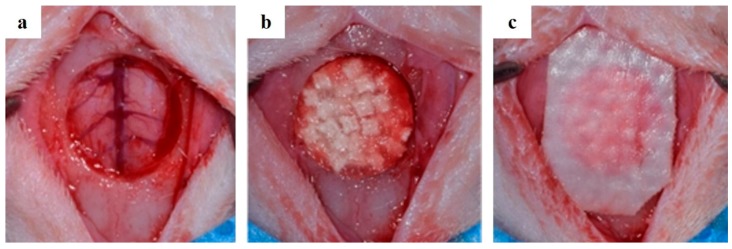
Surgical operation procedures. (**a**) Single defect (8 mm diameter) was formed with a trephine bur; (**b**) The defect area was filled with PCL/PLGA/β-TCP composite block bone grafts; (**c**) The defect area was covered with a collagen membrane.

A mixture of Xylazine (Rumpun, Bayer Korea, Seoul, Korea) and Tiletamin-zolazepam (Zolethyl, Vibac Laboratories, Carros, France) was injected intramuscularly, and general anesthesia was performed. After depilation of the surgical site of the cranium, it was disinfected with betadine and locally anesthetized with 2% lidocaine HCL (Yu-Han Co., Gunpo, Korea) including 1:100,000 epinephrine, after then an incision was performed about 2 cm along the midline. After removal of the periosteum, a circular defect with a diameter of 8 mm was created with a trephine bur (3i Implant Innovation, Palm Beach Garden, FL, USA) (Figure 3).

BCP group implanted 0.125 mg of BCP (Bio-C, Cowellmedi Implant, Seoul, Korea), which was a mixture of HA and β-TCP (3:7 ratio; Ca/P ratio 1.55), to bone defects. The amount of BCP was measured using a micro-spoon (Karl Hammacher, Solingen, Germany) in an amount similar to the average weight of PCL/PLGA/β-TCP particulate bone grafts used in PCL/PLGA/β-TCP composite block bone grafts. The PCL/PLGA/β-TCP group were implanted PCL/PLGA/β-TCP composite block bone grafts, and all groups were covered with collagen membrane (GENOSS, Suwon, Korea).

The periosteum was sutured with the 4-0 absorbable suture (Vicryl^®^, Ethicon, Somerville, NJ, USA), and the skin was layered and sutured using the 4-0 non-absorbable suture. BCP group and PCL/PLGA/β-TCP group were sacrificed by CO_2_ gas at two and eight weeks after surgery.

### 2.7. Histomorphometric Analysis

After sacrifice, the specimen containing the bone graft was fixed with neutral formalin solution (Sigma Aldrich, St. Louis, MO, USA) for two weeks and then removed calcium using the EDTA solution (10%, pH 7.0) after cleaning by distilled water. After calcium removal was confirmed, the ethanol concentration was increased for dehydration. Then, dealcoholization, paraffin infiltration, and paraffin embedding were performed in order. The paraffinized specimen block was sectioned longitudinally in the center of each defect using a microtome, and then mounted on the slide. The thickness of the slides produced was 4 μm. Hematoxylin-eosin (H-E) staining and Masson’s trichrome staining were performed to observe the newly regenerated bone tissues. The most central area was selected from each block for histologic and histomorphometric evaluation. The images on selected slides were saved using an optical microscope connected to a computer (BX51, OLYMPUS, Tokyo, Japan) and a CCD camera (SPOT Insight 2Mp scientific digital camera system, DIAGNOSTIC Instruments Inc., Sterling Heights, MI, USA). The saved images were analyzed by i-Solution ver. 8.1 (IMT i-Solution, Inc., Coquitlam, BC, Canada). Typical specimen images were observed at ×12.5 magnification. For histomorphometric analysis, ×40 and ×400 magnifications were used. New bone area percentage (%) in the defect was analyzed and recorded.

### 2.8. Statistical Analysis

In order to investigate the time-dependent changes and the amount of new bone in each group, SPSS ver. 20 (SPSS Inc., Chicago, IL, USA) was used for statistical analysis. It compared the difference in the amount of new bone in each group through Mann-Whitney test. Statistics were verified at a 5% significance level.

## 3. Results

### 3.1. Components Analysis

**Figure 4:**
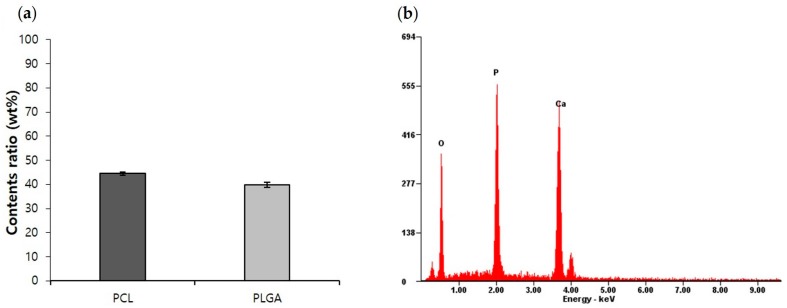
(**a**) Components analysis result for PCL/PLGA/β-TCP particulate bone grafts (measurement of PCL and PLGA components ratio using FT-IR; (**b**) EDS spectrum of β-TCP in PCL/PLGA/β-TCP particulate bone graft.

The actual amounts of PCL and PLGA in fabricated PCL/PLGA/β-TCP particulate bone grafts were measured by comparing the spectrum of fabricated bone grafts–chloroform solution with standard linear curve. Measured amounts of PCL and PLGA were matched (PCL = 44.4 ± 0.69 wt %, PLGA = 39.81 ± 1.06 wt %) with those of raw blended PCL/PLGA/β-TCP (Figure 4a). The result confirmed that the ratio between PCL and PLGA remained the same in the product as intended. In the EDS spectrum of filtered powder in 1 wt % PCL/PLGA/β-TCP particulate bone grafts-chloroform solution, the atomic ratio of Ca/P was 1.38, similar to the theoretical Ca/P value of β-TCP of 1.5 (Figure 4b). Thus, β-TCP was not denatured during blending and the 3D printing process.

### 3.2. SEM Observation

**Figure 5:**
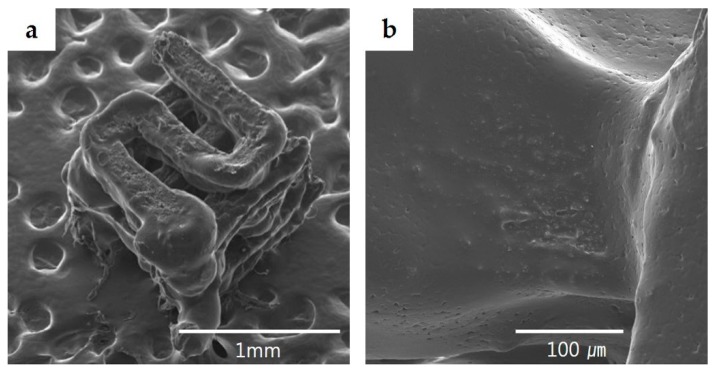
SEM images of PCL/PLGA/β-TCP particulate bone grafts. (**a**) Well-defined PCL/PLGA/β-TCP particulate bone grafts were confirmed at a magnification of ×100; (**b**) Rough surface of PCL/PLGA/β-TCP particulate bone grafts were observed at a magnification of ×800.

The shape and pore structure of micrometer sized PCL/PLGA/β-TCP particulate bone grafts fabricated with SFF technique were confirmed by FE-SEM (Figure 5a). Due to the addition of β-TCP, rough surfaces of PCL/PLGA/β-TCP particulate bone grafts were observed (Figure 5b).

### 3.3. Histological Analysis

**Figure 6:**
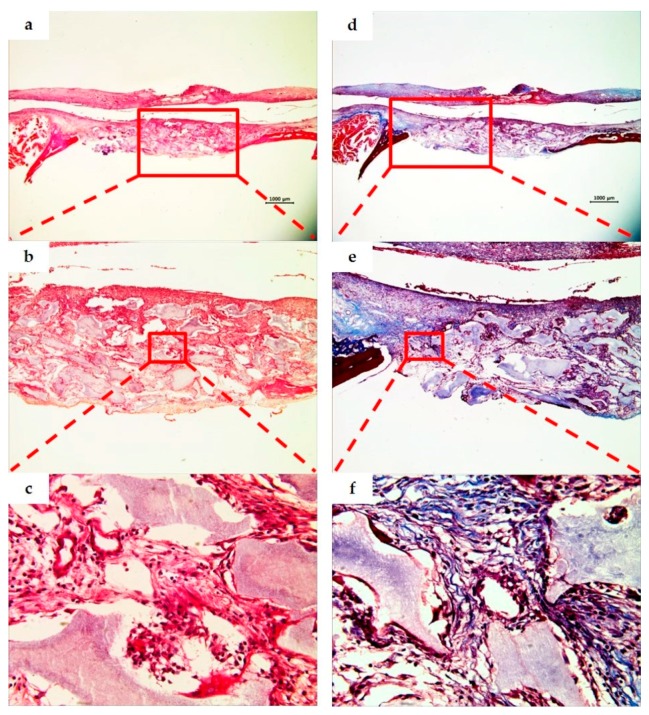
BCP group. Histological sections of defect sites at two weeks after surgery (H&E stain; **a**–**c**. Masson’s trichrome stain; **d**–**f**). Original magnification: ×12.5 (**a**,**d**), ×40 (**b**,**e**), ×400 (**c**,**f**). A large quantity of fibrous connective tissue was formed in the space between the bone grafts, and an immature small amount of new bone was observed around the bone grafts and adjacent bone defect.

**Figure 7:**
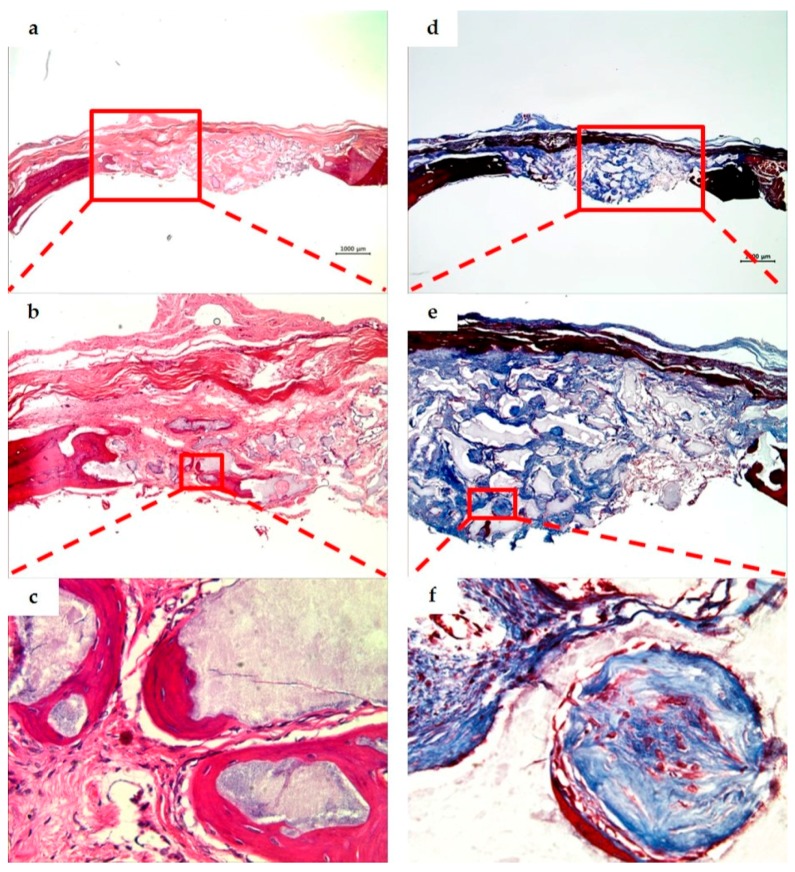
BCP group. Histological sections of defect sites at eight weeks after surgery (H&E stain; **a**–**c**. Masson’s trichrome stain; **d**–**f**). Original magnification: ×12.5 (**a**,**d**), ×40 (**b**,**e**), ×400 (**c**,**f**). The new bone volume and mature bone morphology at eight weeks were observed as being greater than those at two weeks (Figure 6).

As a result of observing the tissue specimens of the BCP group, a large amount of fibrous connective tissue was formed in the space between bone grafts at two weeks, and a small amount of immature new bone was observed around the bone grafts and adjacent bone defect. The particle size of the residual bone grafts varied and showed an irregular distribution throughout the defect. At eight weeks, the amount of new bone was greater than at two weeks, and more mature bone morphology was observed (Figure 6 and Figure 7). 

**Figure 8:**
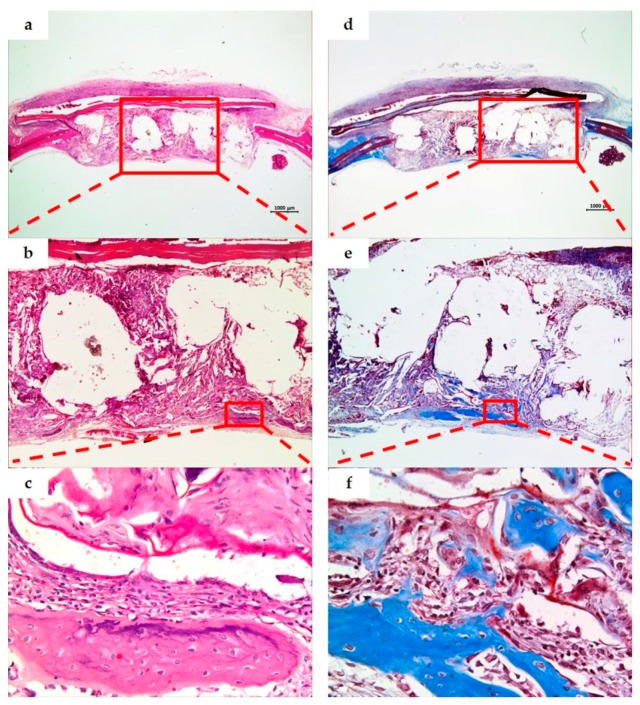
PCL/PLGA/β-TCP group. Histological sections of defect sites at two weeks after surgery (H&E stain; **a**–**c**. Masson’s trichrome stain; **d**–**f**). Original magnification: ×12.5 (**a**,**d**), ×40 (**b**,**e**), ×400 (**c**,**f**). The space of the bone grafts was observed due to demineralization, and the fibrous connective tissue was observed around the bone grafts. New bone formation was limited, and giant cells and inflammatory cell infiltration were found.

**Figure 9:**
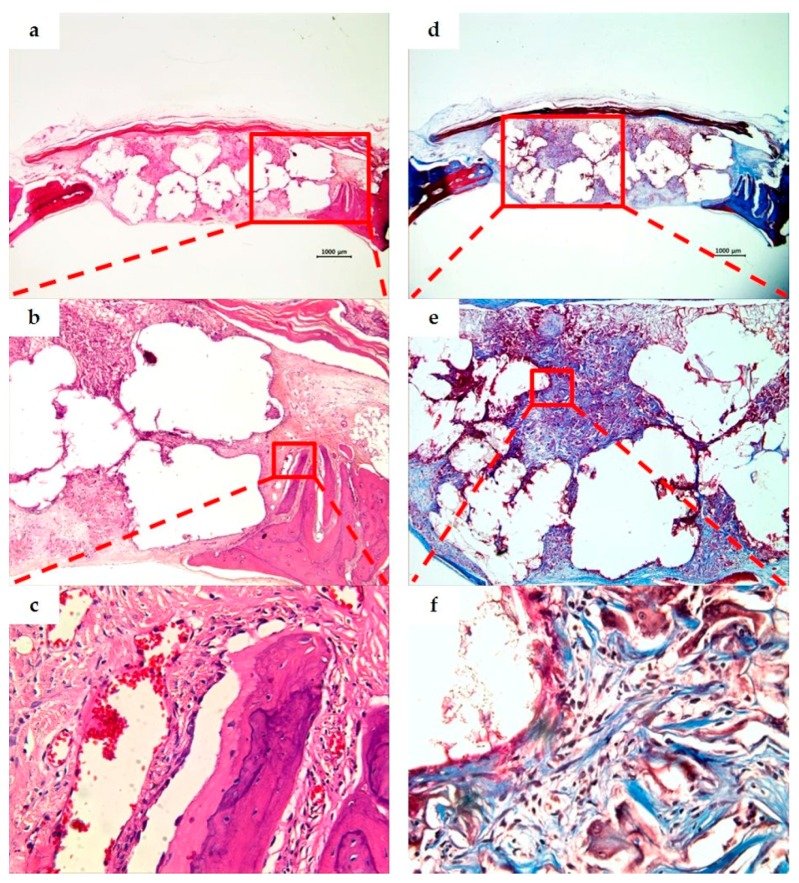
PCL/PLGA/β-TCP group. Histological sections of defect sites at eight weeks after surgery (H&E stain; **a**–**c**. Masson’s trichrome stain; **d**–**f**). Original magnification: ×12.5 (**a**,**d**), ×40 (**b**,**e**), ×400 (**c**,**f**). When compared with the two weeks, more mature peripheral bone tissue was observed, and neovascularization and osteogenesis were observed around the bone grafts. No inflammation was seen.

Tissue specimens of the PCL/PLGA/β-TCP group at two weeks displayed a space formed after PCL/PLGA/β-TCP particulate bone grafts were removed by demineralization, and the particle size was bigger and more uniform than the BCP group. Fibrous connective tissues were formed around the bone grafts, new bone formation was subtle, and giant cell and inflammatory cell infiltration were observed. At eight weeks, the surrounding bone tissue was more mature than at two weeks while neovascularization and new bone formation were observed around the bone grafts. There was no evidence of inflammation. When compared to BCP group, PCL/PLGA/β-TCP group presented unabsorbed bone graft materials and showed excellent space maintenance ability on defects (Figure 8 and Figure 9).

### 3.4. Histometric Analysis

**Figure 10:**
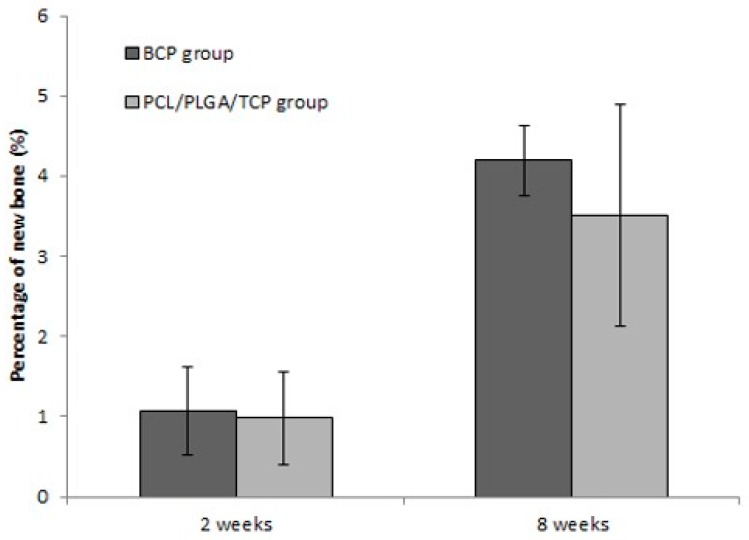
Mean percentage distribution of new bone in defects grafted with the two different bone grafts. At two and eight weeks, there was no significant difference in the amount of new bone between the BCP group and the PCL/PLGA/β-TCP group.

The average and standard deviation of the amount of new bone by each group and period are shown in Table 1. The amounts (%) of new bone in average (±SD) of BCP group were 1.07 (±0.55) and 4.19 (±0.59) at two and eight weeks respectively, and the amounts (%) of new bone in average (±SD) of PCL/PLGA/β-TCP group were 0.98 (±0.43) and 3.51 (±1.38) at two and eight weeks, respectively. There was no significant difference in the amounts of new bone between the PCL/PLGA/β-TCP group and the BCP group at two and eight weeks after applying grafts to the rat calvarial defects (Figure 10).

## 4. Discussion

This study was conducted to compare bone formation ability of PCL/PLGA/β-TCP composite block bone grafts made via SFF technique to BCP which is widely used in the clinical practice. As a result, the amount of new bone formation in PCL/PLGA/β-TCP composite block bone grafts was not significantly different from that in BCP implantation, but the space maintenance ability was better than BCP graft. The requirements for an ideal bone graft material include rapid osteogenesis, promotion of re-vascularization, support of new bone space, biocompatibility, and adequate absorption rate [7,18]. Although the best bone graft material satisfying these requirements is autogenous bone, there are various disadvantages such as additional operation for harvesting, and only a limited amount of bone graft material is available [4,5,6]. Herein, studies on bone graft materials that can replace autogenous bone have been actively conducted [2,4,5,6]. In recent years, 3D printing technologies have been attracting attention in the field of tissue engineering [15,16]. In this study, bone graft materials were fabricated using multi-head deposition system (MHDS) among 3D printing techniques. In MHDS, polymers are melted by heating without using toxic solvents, and pore sizes and fiber thickness can be effectively controlled. [16,19].

Representative synthetic polymeric materials that can be used for SFF technology include PCL and PLGA [14,19]. Among these materials, PLGA is used for regeneration of various tissues due to high biocompatibility [14,19]. However, in the case of a material composed of PLGA alone, it is difficult to maintain shape due to its weak mechanical strength and rapid degradation [14,16]. In contrast, PCL has a degradation rate that is slower than the rate of bone regeneration. In addition, it has excellent mechanical properties and can be used to maintain structure [14,16,20]. Therefore, by mixing PCL and PLGA, bone grafts with superior biologic and mechanical advantages can be produced by complementing weaknesses of each other [14,16,17]. On the other hand, bone grafts made of only ceramic materials such as β-TCP or HA have excellent osteoconductivity, but they can be easily broken due to brittleness of the materials [5]. Therefore, several studies have attempted to produce bone grafts with excellent osteoconductivity and biocompatibility by mixing β-TCP to PCL and PLGA mixture [5,21]. However, such previous studies customized a block-form bone graft based on CT data, requiring much time and effort. In this study, micrometer size particulate bone grafts were prepared by mixing PCL, PLGA, and β-TCP. In order to compensate for the disadvantage of particulate graft materials, which is the difficulty in maintaining shape, collagen matrix was added to make composite block bone grafts.

Collagen is the main structural protein for tissue support and remodeling upon recovery from physical trauma [22]. It also plays an important role in providing biological support for cellular activities associated with cell attachment, migration, and differentiation [22]. Collagen is a tropocollagen structure that is not soluble in its natural state, and although it has low antigenicity in this state, the telopeptide component at the N and C terminal of tropocollagen can act as a heterologous antigen [23]. To solve this problem, telopeptide of both ends was removed by using enzyme pepsin and processed into atelocollagen [22]. Atelocollagen has advantages of excellent biocompatibility, lower molecular weight, and faster absorption rate than collagen [24]. Composite block bone graft materials based on collagen have excellent biocompatibility and stretchability, but it is necessary to combine hard materials such as inorganic particles due to their weak mechanical strength [12]. Among the previous attempts, the composite block bone graft using bovine bone particles showed excellent new bone conduction and space maintenance, but the bovine bone particles had low biodegradability which was a disadvantage wherein they remained in the new bone tissue for a long time. [12,25]. The previous studies on composite block-type bone graft materials with β-TCP particles showed excellent osteoconductivity and adequate degradation rate, but the mechanical strength was low [12,26]. In this study, bone graft materials were prepared using PCL and PLGA, which have excellent biodegradability and mechanical properties.

The mechanical properties and degradation rate of synthetic bone grafts may vary depending on composition ratio of each material [27]. Stiffness is increased with an increase in PLGA ratio [27,28]. In the previous study in which the barrier membrane was fabricated with PCL/PLGA/β-TCP at a ratio of 2:6:2, the stiffness was similar to that of titanium membrane [27,28]. In this study, the ratio of PCL/PLGA/β-TCP was set to 4:4:2, which increased the proportion of PCL with flexible properties and improved the operability and formability [21]. In addition, it can maintained a proper shape in the defect site and is considered to be superior to the BCP group in terms of space maintenance for bone regeneration. The space maintenance ability of bone grafts that can support barrier membrane for implant or periodontal operations is an essential factor [18]. This can be avoided by the collapse of immature soft tissue or the growth of soft tissues on teeth or implants [18]. In this study, structural integrity was successfully restored upon PCL/PLGA/β-TCP composite block bone graft application as it was before forming the rat calvarial defects. PCL/PLGA/β-TCP composite block bone grafts were useful in supporting barrier membranes. In contrast, in the BCP group, the collapse of the existing shape and the distribution of irregular grafts have been shown in the recovery phase. When considering that the mechanism of osteogenesis of synthetic bone is osteoconduction, volume stability that can act as a scaffold until new bone formation is an important factor.

In the present study, we compared the bone regeneration ability of PCL/PLGA/β-TCP composite block bone graft made by 3D printing to that of BCP, which is a synthetic bone graft mainly used in clinical practice. In PCL/PLGA/β-TCP group, a lot of collagen was produced, which led to the active proliferation of surrounding cells and blood vessels as well as cell migration at two weeks. At eight weeks, through bone conduction process, we could observe bone remnants formed from the adjacent bone. In histomorphometric analysis, there was no significant differences in the amount of new bone between the PCL/PLGA/β-TCP group and BCP groups. In this experiment, the amount of bone formation around the bone grafts was lower compared to the amount of bone formation from the defect boundary in the PCL/PLGA/β-TCP group. It might be due to the improper absorption rate of bone grafts. The rate of absorption of bone grafts should be similar to that of new bone replacement. Many studies have reported that, in case of a faster rate of absorption, the supporting ability gets weaker while in a slower rate of absorption it may interfere with the formation of new bone [12,29,30]. The factors affecting bioabsorbability of bone grafts include their shape, size, surface area, and porosity [31]. Generally, it is known that the larger particle size may result in a longer residual period in vivo since it could interfere with new bone formation [32]. It has also been reported that higher bone formation rate was observed in the case of smaller particle sizes and larger porosity in BCP with similar composition [20,33]. In this study, the PCL/PLGA/β-TCP composite block bone grafts applied to the rat calvarial defect had a relatively large particle size, so it would be better if the particle size was reduced. In addition, collagen matrix used in fabricating composite block bone grafts may have affected new bone formation. In a previous study of composite block bone grafts based on polysaccharides, such as carboxymethyl cellulose (CMC) and hyaluronic acid, higher new bone formation was reported in BCP mixed with crosslinked CMC compared to the particle type BCP [34]. In the future, further studies using various types of matrix such as polysaccharides will be needed for better bone regeneration. Furthermore, the optimal shape, size, and composition of the bone grafts needs to be determined, and growth factors such as bone morphogenic proteins can also be applied to promote bone growth.

## 5. Conclusions

Although 3D printed PCL/PLGA/β-TCP composite block bone grafts were not superior in bone regeneration ability compared to the conventional BCP, the results showed their relatively similar performances. Therefore, PCL/PLGA/β-TCP composite block bone grafts have potential to be applied in synthetic bone grafts clinically.

## Figures and Tables

**Table 1:** Histomorphometric analysis (mean ± SD).

Groups	2 Weeks		8 Weeks	
	n	NB (%) ^a^	n	NB (%) ^a^
BCP	8	1.07 ± 0.55	8	4.19 ± 0.59
PCL/PLGA/β-TCP	8	0.98 ± 0.43	8	3.51 ± 1.38
*p*-value ^b^		0.674		0.345

^1^ Newly formed bone. ^2^ *p*-values are computed by Mann-Whitney test.

## Abbreviations

The following abbreviations are used in this manuscript:

3D	Three-dimensional
PCL	Polycaprolactone
PLGA	Poly (lactic-co-glycolic acid)
β-TCP	β-tricalcium phosphate
BCP	Biphasic calcium phosphate
EDS	Energy dispersive X-ray spectroscopy
SFF	Solid freeform fabrication
SEM	Scanning electron microscope
FE-SEM	Field emission scanning electron microscope
MHDS	Multi-head deposition system
EDC	1-ethyl-3-(3-dimethyaminopropyl) carbodiimide
NHS	N-hydroxysuccinimide
